# Searching for Preclinical Models of Acute Decompensated Heart Failure: a Concise Narrative Overview and a Novel Swine Model

**DOI:** 10.1007/s10557-020-07096-5

**Published:** 2020-10-24

**Authors:** Davide Olivari, Daria De Giorgio, Lidia Irene Staszewsky, Francesca Fumagalli, Antonio Boccardo, Deborah Novelli, Martina Manfredi, Giovanni Babini, Anita Luciani, Laura Ruggeri, Aurora Magliocca, Davide Danilo Zani, Serge Masson, Angelo Belloli, Davide Pravettoni, Giuseppe Maiocchi, Roberto Latini, Giuseppe Ristagno

**Affiliations:** 1 Department of Cardiovascular Medicine, Istituto di Ricerche Farmacologiche Mario Negri IRCCS, via Mario Negri 2, 20156 Milan, Italy; 2 DIMEVET, Veterinary Teaching Hospital, University of Milan, Lodi, Italy; 3 Novartis Farma Spa, Origgio, VA Italy; 4 Department of Pathophysiology and Transplantation, University of Milan, via Francesco Sforza 35, 20122 Milan, Italy

**Keywords:** Acute heart failure, Congestion, Pulmonary edema, Swine

## Abstract

**Purpose:**

Available animal models of acute heart failure (AHF) and their limitations are discussed herein. A novel and preclinically relevant porcine model of decompensated AHF (ADHF) is then presented.

**Methods:**

Myocardial infarction (MI) was induced by occlusion of left anterior descending coronary artery in 17 male pigs (34 ± 4 kg). Two weeks later, ADHF was induced in the survived animals (*n* = 15) by occlusion of the circumflex coronary artery, associated with acute volume overload and increases in arterial blood pressure by vasoconstrictor infusion. After onset of ADHF, animals received 48-h iv infusion of either serelaxin (*n* = 9) or placebo (*n* = 6). The pathophysiology and progression of ADHF were described by combining evaluation of hemodynamics, echocardiography, bioimpedance, blood gasses, circulating biomarkers, and histology.

**Results:**

During ADHF, animals showed reduced left ventricle (LV) ejection fraction < 30%, increased thoracic fluid content > 35%, pulmonary edema, and high pulmonary capillary wedge pressure ~ 30 mmHg (*p* < 0.01 vs. baseline). Other ADHF-induced alterations in hemodynamics, i.e., increased central venous and pulmonary arterial pressures; respiratory gas exchanges, i.e., respiratory acidosis with low arterial PO_2_ and high PCO_2_; and LV dysfunction, i.e., increased LV end-diastolic/systolic volumes, were observed (*p* < 0.01 vs. baseline). Representative increases in circulating cardiac biomarkers, i.e., troponin T, natriuretic peptide, and bio-adrenomedullin, occurred (*p* < 0.01 vs. baseline). Finally, elevated renal and liver biomarkers were observed 48 h after onset of ADHF. Mortality was ~ 50%. Serelaxin showed beneficial effects on congestion, but none on mortality.

**Conclusion:**

This new model, resulting from a combination of chronic and acute MI, and volume and pressure overload, was able to reproduce all the typical clinical signs occurring during ADHF in a consistent and reproducible manner.

**Electronic supplementary material:**

The online version of this article (10.1007/s10557-020-07096-5) contains supplementary material, which is available to authorized users.

## Introduction

Acute heart failure (AHF) is characterized by a gradual or rapid onset of new or worsening signs and symptoms of heart failure (HF), usually requiring hospitalization for urgent therapy. Indeed, AHF is the most common cause for hospital admission in patients over 65 years, with approximately 1 million hospitalizations per year in the USA and Europe, and a high burden of costs, i.e., over $60 billion/year [1]. Despite improvements in the management of HF patients, the prognosis after hospitalization for AHF remains bleak, with rates of death or recurrent hospitalization at 6 months approaching 50% [2].

Lung congestion leading to dyspnea is the main reason for hospitalization in patients with acute decompensated heart failure (ADHF). Although, the majority of patients respond readily to loop diuretics, achieving complete decongestion can be challenging, so that a significant proportion (> 30%) are discharged with persistent evidence of congestion. Residual congestion is associated with a high risk of early rehospitalization and death. Among patients discharged from the hospital for ADHF, only 32.9% are alive and have not been rehospitalized after 1 year [3]. Thus, widespread efforts to develop new drug treatments, as well as to develop robust preclinically animal models to test therapeutic interventions, are highly motivated [2–5].

Over the years, several animal models have been employed in order to discover disease mechanisms and to develop new therapies for AHF [6–14]. Among them, the porcine model has been increasingly used due to its similarities to the human cardiac pathophysiology and genetics [8–13]. Although different approaches have been used and reported to reproduce experimentally the HF condition, they all present important limitations, duplicating AHF only partially. The need to develop new in vivo models able to accurately resemble the clinical traits of AHF is of pivotal importance in order to test the safety and efficacy of new drugs prior to being clinically translated. In addition, there is still the lack of a valid model reproducing specifically the condition of ADHF [6, 10–12, 14].

Thus, the aim of the present study was to set up a novel, highly reproducible, and preclinically relevant porcine model to mimic the common pathophysiological clinical condition observed in ADHF patients [2, 3, 15, 16]. Continuous infusion of serelaxin, recombinant human relaxin-2 investigated recently as therapeutic agent in congestive HF patients [17–20], was also performed with the intent to validate the proposed model in studying the effects of new experimental drugs.

## Materials and Methods

### Ethics

All procedures involving animals and their care were in conformity with national and international laws and policies (Legislative Decree n° 76/2014-B). Approval of the study was obtained by the governmental review board committee and governmental institution (Italian Ministry of Health, approval no. 83/2014-PR).

For the study, 17 male *Sus scrofa domesticus* pig weighing 34 ± 4 kg were used. Animals were obtained from the same breeder and were housed in the animal facility of the Veterinary Teaching Hospital, University of Milan, Lodi, Italy. Pigs were housed in pairs before the experiment, and individually after, under controlled temperature, humidity, and illumination condition, and with food and water being available ad libitum. Environmental enrichment was used. Animals were allowed a minimum of 4 days to adapt to housing conditions before undergoing any manipulation.

### Experimental Design

**Fig. 1:**
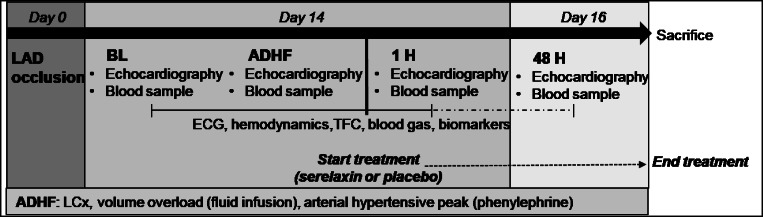
Experimental design. LAD, left anterior descending coronary artery; BL, baseline; ADHF, acute decompensated heart failure; LCx, left circumflex coronary artery; TFC, thoracic fluid content; H, hours post-ADHF

The experimental design is detailed in Fig. 1. On day 0, pigs were anesthetized, and acute myocardial infarction (AMI) was induced by occlusion of left anterior descending (LAD) coronary artery. Two weeks later (day 14), the survived animals were re-anesthetized and surgically instrumented for invasive hemodynamics monitoring. ADHF was then induced by occlusion of the circumflex coronary artery (LCx), associated with acute volume overload and increase in arterial blood pressure by vasoconstrictor infusion. After onset of ADHF, animals were divided to receive continuous iv infusion of either serelaxin or saline placebo (control) over the following 48-h observational period.

### Animal Preparation

Seventeen pigs were fasted the night before the experiment except for free access to water. Anesthesia was induced by intramuscular injection of ketamine (20 mg/kg) followed by intravenous administration of propofol (2 mg/kg) and sufentanil (0.3 μg/kg) through an ear vein access. Anesthesia was then maintained by continuous intravenous infusion of propofol (4–8 mg/kg/h) and sufentanil (0.3 μg/kg/h). A cuffed tracheal tube was placed, and animals were mechanically ventilated with a tidal volume of 15 mL/kg and FIO_2_ of 0.21 (Bellavista 1000, IMT, Switzerland). Respiratory frequency was adjusted to maintain the end-tidal PCO_2_ (EtCO_2_) between 35 and 40 mmHg, monitored with an infrared capnometer (Philips MRx, USA) [21, 22]. To measure aortic pressure, a fluid-filled 7F catheter was advanced from the right femoral artery into the thoracic aorta. For right atrial pressure (RAP), pulmonary arterial pressure (PAP), pulmonary capillary wedge pressure (PCWP), core temperature, and cardiac output (CO) measurements, a 7F pentalumen thermodilution catheter was advanced from the right femoral vein into the pulmonary artery. Conventional pressure transducers were used (MedexTransStar, Monsey, NY). The position of all catheters was confirmed by characteristic pressure curve morphology and/or by digital fluoroscopy. A frontal plane electrocardiogram (ECG) was recorded. Temperature of the animals was maintained at 38 °C ± 0.5 °C during the whole procedure [21, 22].

### AMI Induction

First AMI was induced in a closed-chest preparation by intraluminal occlusion of the LAD. A 6F balloon-tipped catheter (Edwards Lifesciences, Italy) was inserted from the right common carotid artery and advanced into the aorta, then into the LAD, beyond the first diagonal branch, with the aid of image intensification, and confirmed by injection of radiographic contrast media (Visipaque®-320, GE Healthcare, Italy) [21, 22]. The balloon of the LAD catheter was then inflated with 0.8 mL of air to occlude the flow. The LAD was maintained occluded for 1 h, followed by reperfusion. The catheter was then removed, the wound was repaired, and the animals were extubated and returned to their cages for 2 weeks prior to inducing ADHF. Analgesia with butorphanol (0.1 mg/kg) was given by intramuscular injection.

### ADHF Induction and Treatment

Two weeks after the first AMI, a second AMI was induced in the survived animals (*n* = 15) by occluding the LCx through the same closed-chest preparation procedure described for the LAD. Volume overload was then induced by infusing iv a large volume of crystalloids (ringer acetate and saline) over 1 h, and an arterial hypertensive peak (pressure overload) was concurrently induced by iv infusion of phenylephrine (50 μg, total dose).

Animals were considered in ADHF when all the following three criteria were met: (1) a sustained left ventricular ejection fraction (LVEF) < 30%, (2) a 3-fold increase in PWCP, and (3) an increase of thoracic fluid content (TFC) of more than 25% from the baseline value.

After ADHF occurred, the LCx balloon was deflated, the catheter was removed, and crystalloids and phenylephrine infusion was stopped. An Alzet® osmotic pump (CA, USA) was implanted for a continuous iv infusion of either serelaxin (30 μg/kg/day, *n* = 9) or saline (*n* = 6) for the following 48 h. The pump was surgically positioned and secured with sutures between the right sternocleidomastoid muscle bands; the polyethylene catheter was inserted into the right external jugular vein and advanced for 8 cm (Supplemental Figure).

Animals were maintained under anesthesia and invasively monitored for 1 h. Catheters were then removed, wounds were sutured, and the animals were extubated and returned to their cages. Analgesia was performed with butorphanol (0.1 mg/kg) given by intramuscular injection. Animals underwent daily clinical examination by veterinarian doctors to identify and eventually alleviate signs of severe pain, severe distress, or suffering, i.e., poor physical appearance, severe respiratory distress, neurological signs, rapidly progressive weight loss, and impending death. Under judgment of the responsible veterinary doctor, termination of the experiment could be considered by humanely euthanizing the animal (this instance, however, did not occur). At the end of the 48-h treatment, animals were re-anesthetized for hemodynamic measurements as described above (using the left femoral artery and vein accesses), echocardiographic examination, TFC quantification, and blood sample withdrawal. The pigs were then euthanized painlessly with an intravenous injection of 150 mg/kg sodium thiopental, and the heart and lung were harvested [21, 22].

### Measurements

Hemodynamics, heart rate (HR), and EtCO_2_ were recorded continuously on a personal computer–based acquisition system (WinDaq DATAQ Instruments Inc., Akron, OH). CO was measured by using the thermodilution technique (COM-2; Baxter International Inc., Deerfield, IL) [21, 22].

To quantify lung congestion, TFC was continuously measured by non-invasive chest bioimpedance. Chest bioimpedance was assessed through four skin sensors placed on the neck and left side of the thorax to allow for the continuous measurement of the changes of electrical conductivity within the thorax with the aid of an ICON electrical cardiometer (Cardiotronic Osypka Medical, Germany).

Left ventricular (LV) function was echocardiographically evaluated during the different experimental timepoints. Transthoracic echocardiography was performed using a phased-array multifrequency 2.5- to 5-MHz probe (CX50, Philips, Netherlands). LV volumes and ejection fraction calculations (LVEF) were performed on two-dimensional (2D) images obtained from two and four apical views using the modified Simpson’s method of discs. LV diastolic function was evaluated by combined pulse wave and tissue Doppler calculating the early diastolic trans-mitral flow velocity (*E*) and the early diastolic mitral annular tissue velocity (*e′*) ratio (*E*/*e′*) from the four apical chamber views. Echocardiographic recordings and measurements were followed at 5-min intervals from baseline (supplemental video 1: apical 4-chamber view, and supplemental video 2: apical 2-chamber view) until ADHF onset (supplemental video 3: ADHF apical 4-chamber view, and supplemental video 4: ADHF apical 2-chamber view), and then 1 h and 48 h later. For all images, 3–5 cardiac cycles with simultaneous 3-lead ECG signal were recorded, and measurements were followed according to the American and European Societies of Echocardiography Guidelines by a cardiologist blinded to study groups [21–23].

Chest computed tomography (CT) was executed with a 16-slice scanner (GE Brightspeed®, GE Healthcare, Italy) only in one representative animal to visualize the presence of lung congestion.

**Fig. 2:**
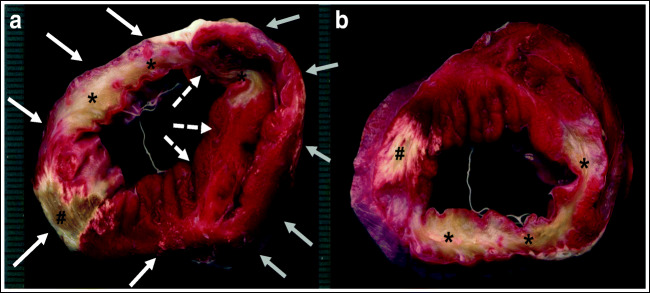
Myocardial infarct size. Triphenyltetrazolium chloride (TTC) staining of the upper (**a**) and lower (**b**) faces of one ventricle slice. Vital myocardium is TTC-positive (red), whereas infarcted myocardium is TTC-negative (white). White arrows: left ventricle wall; dashed arrows: interventricular septum; and gray arrows: right ventricle wall. Two different areas of infarct, from LAD (*) and LCx occlusion (#), are visible

Myocardial infarct size was assessed by triphenyltetrazolium chloride (TTC) staining. The ventricles were sliced into 5-mm-thick transverse sections, which were incubated (20 min) in a solution of TTC and then transferred to 10% neutral buffered formalin overnight before image analysis. Infarct size (deriving from the occlusion of both LAD and LCx) was reported as a percentage of TTC-negative area relative to LV plus septum area, as presented in Fig. 2 [21, 22].

After separating a portion of the caudal left lung lobe of each animal, it was weighed and placed in the oven at 80 °C for ≥ 72 h. After removal from the oven, it was reweighed for calculation of the wet/dry weight ratio, an indicator of lung congestion.

Arterial blood samples were withdrawn at baseline prior to inducing ADHF, at ADHF occurrence, and after 1 h and 48 h of treatment. Arterial blood gasses were assessed with the i-STAT System (Abbott Laboratories, Princeton, NJ). Plasma high-sensitivity cardiac troponin T (hs-cTnT) was measured with an electrochemiluminescence assay (Cobas, Roche Diagnostics, Rotkreuz, CH). N-terminal proatrial natriuretic peptide (NT-proANP) was assayed with a validated ELISA kit (Biomedica BI-20892). Circulating levels of bio-adrenomedullin (Invivo Biotech Service, Germany) were quantified as marker of pulmonary congestion. In order to further investigate renal and liver function, serum levels of creatinine and alanine transferase (ALT) were measured (Colorimetric Activity Assay Kit, Cayman Chemical, USA). Finally, plasmatic levels of relaxin-2 were measured with an ELISA kit (Quantikine ELISA kit Human Relaxin-2, R&D Systems, Minneapolis, MN).

### Statistical Analysis

A one-sample Kolmogorov–Smirnov *Z* test was used to confirm the normal distribution of the data. For comparison of time-based variables, two-way analysis of variance (ANOVA) with Sidak multiple comparison test was performed. A logarithmic (Log_10_) transformation was executed prior to analyzing variables that were not normally distributed. For comparisons of variables with only a timepoint, one-way analysis of variance was used. When the dependent variable was categorical, a Fisher exact test was used. All data are reported as mean ± SEM. A *p* value ≤ 0.05 was regarded as statistically significant. GraphPad Prism 7.5 (GraphPad Software Inc., La Jolla, CA) was used for statistical analyses.

## Results

### ADHF Induction

**Fig. 3:**
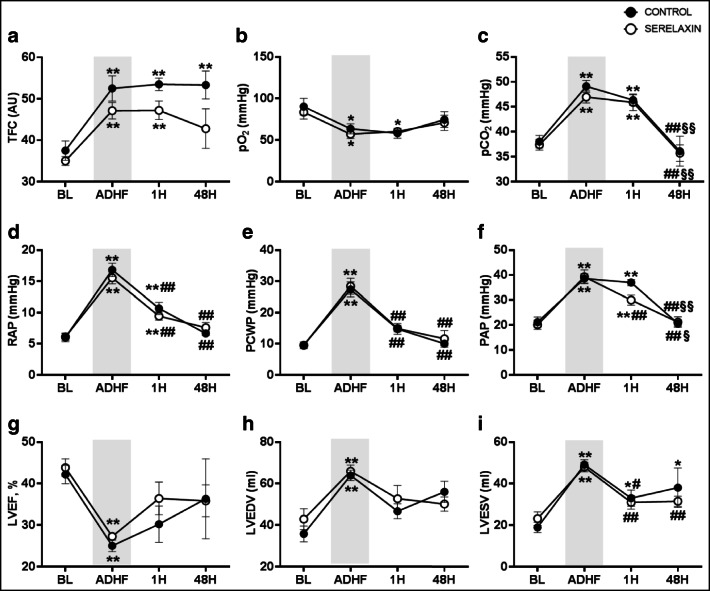
Hemodynamics, gas exchanges, and myocardial function in control and serelaxin-treated animals, at baseline (BL), onset of acute decompensated heart failure (ADHF), and 1 and 48 h (H) later. TFC, thoracic fluid content (A); pO_2_, arterial partial pressure of oxygen (B); pCO_2_, arterial partial pressure of dioxide (C); RAP, right atrial pressure (D); PCWP, pulmonary capillary wedge pressure (E); PAP, pulmonary arterial pressure (F); LVEF, left ventricle ejection fraction (G); LVEDV, left ventricle end-diastolic volume (H); LVESV, left ventricle end-systolic volume (I). All data are reported as mean ± SEM. *p ≤ 0.05 and **p ≤ 0.01 vs. BL; ^#^*p* ≤ 0.05 and ^##^*p* ≤ 0.01 vs. AHF; ^§^*p* ≤ 0.05 and ^§§^*p* ≤ 0.01vs. 1H

**Fig. 4:**
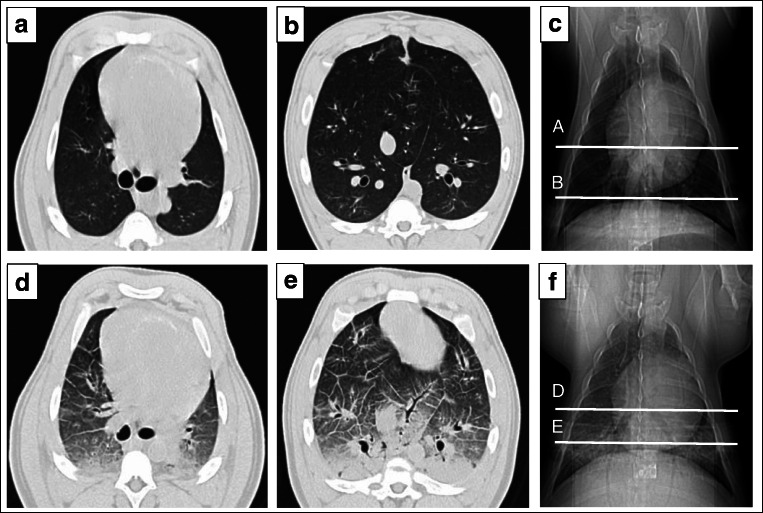
Representative CT images of lungs in a pig prior and after acute decompensated heart failure (ADHF). **a** Cranial lungs prior ADHF, surrounded by the rib cage are lungs (dark gray) and heart (white). Left lung is located at the left of the image, while the right ventricle and the right lung is on the right. White lines that partially fill lungs represent blood vessels. A thick layer of fat and skin envelops the rib cage and the dorsal and abdominal muscles. **b** Caudal lungs prior ADHF. Lungs with blood vessels cover all the area enclosed by the rib cage. From the picture, a division in lobes could be seen (from left to right: left caudal lobe, accessory lobe, right caudal lobe). **c** Chest prior ADHF. Inside the rib cage, the heart (white) and lungs (black). Trachea and primary bronchi look like black under the thoracic spine, whereas blood vessels appear as transparent gray across the lungs. At the bottom, the diaphragm separates the thorax from the abdomen. White lines indicate where the scout cut is to obtain A and B images. **d** Cranial lungs after ADHF. CT scan shows bilateral ground-glass opacity with thickening of peribronchovascular interstitium, interlobular septa, and pleural fissures. **e** Caudal lungs after ADHF. Bilateral pulmonary ground-glass opacity and consolidations with gravity-dependent distribution. Thickening of peribronchovascular interstitium, interlobular septa, and pleural fissures. The apex of the heart is visible at the top of the rib cage. **f** Chest after ADHF. White reverberations can be seen throughout all the lungs’ image, especially at the basal level. White lines indicate where the scout cut is to obtain D and E images

Two animals died due to the first AMI. Thus, ADHF was induced in 15 pigs. The total amount of crystalloids infused was 5.7 ± 0.5 L in the control group and 5.2 ± 0.3 L in the serelaxin one (*p* = 0.43). Criteria for ADHF were achieved in all animals, with a reduction in LVEF < 30% and increases in TFC > 25% from baseline and in PCWP to values of 28 ± 2 mmHg (*p* < 0.01 vs. baseline, Table 1 and Fig. 3). Other typical ADHF-induced alterations in hemodynamics, i.e., increases in RAP and PAP; in respiratory gas exchanges, i.e., respiratory acidosis; and in LV function, i.e., increased left ventricle end-diastolic volume (LVEDV) and left ventricle end-systolic volume (LVESV) and decreased ejection fraction (EF), were observed constantly in all animals (*p* < 0.01 vs. baseline, Fig. 3). The presence of lung congestion was also visualized by CT scan imaging performed in one representative animal (Fig. 4). Median plasmatic levels of relaxin-2 significantly increased in animals receiving continuous 48-h iv infusion of serelaxin, i.e., from 8 at baseline to 2343 and 7714 pg/mL at 1 and 48 h, respectively, compared to controls in which values remained stable, i.e., 46, 24, and 17 pg/mL at baseline, 1 and 48 h, respectively (*p* < 0.05 vs. serelaxin).

**Table 1:** Survival, hemodynamics, pH, and myocardial function

	Control (*n* = 6)	Serelaxin (*n* = 9)
BW, kg	34 ± 1	35 ± 1
48 h survival, *n*/tot	3/6	5/9
Time to death, h	25 ± 13	10 ± 5
HR, b/min		
BL ADHF 1 h 48 h	116 ± 15 97 ± 11 95 ± 7 101 ± 5	112 ± 8 101 ± 8 95 ± 14 122 ± 7
SAP, mmHg		
BL ADHF 1 h 48 h	101 ± 7 119 ± 12 119 ± 6 71 ± 2	107 ± 6 157 ± 17** 120 ± 10^#^ 96 ± 10^##^
MAP, mmHg		
BL ADHF 1 h 48 h	85 ± 7 98 ± 12 104 ± 6 53 ± 4	92 ± 6 133 ± 17* 104 ± 9 71 ± 7^##^
DAP, mmHg		
BL ADHF 1 h 48 h	72 ± 6 78 ± 12 90 ± 6 44 ± 4	82 ± 5 109 ± 16 93 ± 8 60 ± 7^##^
CO, L/min		
BL ADHF 1 h 48 h	2.9 ± 0.4 3.1 ± 0.4 2.1 ± 0.2 3.2 ± 0.5	2.8 ± 0.2 3.7 ± 1.4 2.4 ± 0.3 3.0 ± 0.3
EtCO_2_, mmHg		
BL ADHF 1 h 48 h	35 ± 1 36 ± 1 36 ± 1 36 ± 0	36 ± 1 35 ± 1 36 ± 0 36 ± 0
pH		
BL ADHF 1 h 48 h	7.461 ± 0.021 7.167 ± 0.022** 7.242 ± 0.026** 7.520 ± 0.0^§§##^	7.481 ± 0.017 7.206 ± 0.020^**^ ^7.266 ± 0.036**^ 7.474 ± 0.019^§§##^
*E*/*e′* lateral ratio		
BL 1 h 48 h	11 ± 2 19 ± 4* 9 ± 0^§^	8 ± 1 10 ± 2^++^ 7 ± 1
Infarct size, %	29.7 ± 14.8	21.5 ± 8.8
Lung wet/dry ratio, g	8 ± 0.5	5.9 ± 0.3^++^

*BL*, baseline; *h*, hours; *ADHF*, acute decompensated heart failure; *BW*, body weight; *HR*, heart rate; *SAP*, systolic arterial pressure; *MAP*, mean arterial pressure; *DAP*, diastolic arterial pressure; *EtCO*_*2*_, end-tidal CO_2_; *lateral E/e′*, peak diastolic mitral inflow *E* velocity to tissue Doppler early diastolic excursion of the lateral mitral annulus in the left ventricular lateral wall

All data are reported as mean ± SEM

^*^p ≤ 0.05 and ** *p* ≤ 0.01 vs. BL

^#^*p* ≤ 0.05 and ^**##**^*p* ≤ 0.01 vs. AHF

^§^*p* ≤ 0.05 and ^§§^*p* ≤ 0.01vs. 1 h

^++^*p* ≤ 0.01 vs. control

With approximately 50% of animals surviving up to 48 h, no difference in mortality was observed between the two groups, although a trend towards earlier death was observed in the serelaxin group compared to the control one (*p* = 0.25, Table 1).

### Lung Congestion and Respiratory Gas Exchanges

TFC increased 40% in the control group and 35% in the serelaxin one compared to baseline at onset of ADHF (*p* < 0.01 vs. baseline, Fig. 3a). TFC value remained high in the control group 48 h later, while it decreased in the animals that received serelaxin. Overall, TFC was lower in animals treated with serelaxin compared to controls (*p* = 0.0015).

Arterial blood gasses showed an important systemic acidosis, with significantly reduced pH and arterial pO_2_ and significantly increased arterial pCO_2_ at onset of ADHF compared to baseline (*p* < 0.01 vs. baseline, Table 1 and Fig. 3b and c).

### Hemodynamics

RAP and PAP increased significantly compared to baseline (*p* < 0.01, Fig. 3d and f) during ADHF in both groups, as expected, and then recovered 48 h later. In animals receiving serelaxin, PAP started to significantly decrease already after 1 h of treatment (*p* < 0.01 vs. AHF). PCWP markedly increased in both groups after the onset of ADHF (*p* < 0.01 vs. baseline, Fig. 3e), to then recover in the following hour. HR numerically decreased in all animals during AHF (*p* = 0.53). Forty-eight hours later, HR increased in animals that received serelaxin, while it remained low in controls (*p* = 0.77, Table 1). Arterial pressure increased at onset of ADHF compared to baseline, due to vasoconstrictor infusion, but then decreased significantly 48 h later (Table 1).

### Echocardiography

Baseline LVEF was low in all animals due to the AMI episode that occurred 2 weeks before. At ADHF onset, mean LVEF decreased to 25% in the control group and to 27% in the serelaxin one (*p* < 0.01 vs. baseline, Fig. 3g). One and 48 h later, LVEF increased, although values remained lower than baseline (*p* = 0.0613 and *p* = 0.7321 for 1 and 48 h, respectively, in control; *p* = 0.24 and *p* = 0.29 for 1 and 48 h, respectively, in serelaxin). Similarly, LVEDV and LVESV significantly increased compared to baseline in both groups (*p* < 0.01 vs. baseline, between groups, Fig. 3h and i, and Supplemental Videos 1–4). LVEDV values remained high during the 48 h of observation in both groups (Fig. 3h). LVESV decreased significantly already 1 h after ADHF, but values remained higher compared to baseline, especially in the serelaxin group (*p* < 0.05 vs. baseline, *p* = 0.98 vs. control, Fig. 3i). *E*/*e′* lateral ratio did not change significantly in the serelaxin group overtime, while it increased significantly in the control one 1 h after ADHF onset (Table 1).

### Circulating Biomarkers

**Fig. 5:**
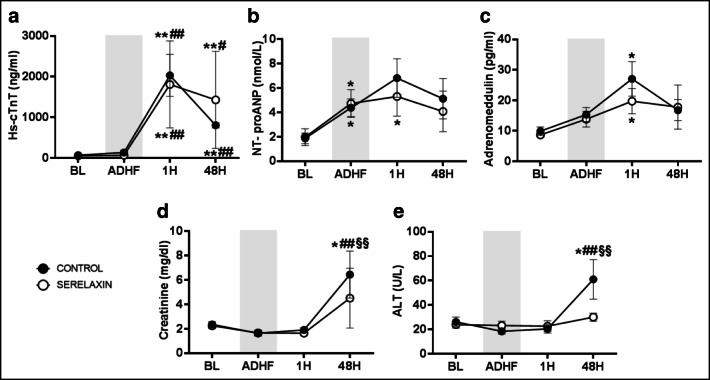
Circulating biomarkers in control and serelaxin-treated animals, at baseline (BL), onset of acute decompensated heart failure (ADHF), and 1 and 48 h (H) later. Hs-cTnT, high sensitive cardiac troponin T (A); NT-proANP, n-terminal proatrial natriuretic peptide (B), creatinine (C); ALT, alanine aminotransferase (D); adrenomedullin (E). All data are reported as mean ± SEM. *p ≤ 0.05 and **p ≤ 0.01 vs. BL; ^##^*p* ≤ 0.01 vs. AHF; ^§§^*p* ≤ 0.01vs. 1H

Plasma hs-cTnT levels increased significantly 1 h after ADHF onset and remained high 48 h later (*p* < 0.01 vs. baseline, Fig. 5a). Plasma NT-proANP, instead, increased significantly already at the time of AHF onset (*p* < 0.05 vs. baseline, Fig. 5b). Plasmatic levels of bio-adrenomedullin increased 1 h after the ADHF onset (*p* < 0.05 vs. baseline) and started to decrease at 48 h (Fig. 5c). Biomarkers of renal and liver injuries increased significantly from baseline values only in the control group 48 h after ADHF (*p* < 0.01 vs. baseline, Fig. 5d and e).

### Histopathology

At histopathology, no difference in myocardial infarct size was observed between the two groups (Table 1), while the lung water content assessed as the wet-to-dry ratio was significantly lower in animals treated with serelaxin compared to controls (*p* < 0.01, Table 1).

## Discussion

This new model of ADHF, resulting from a combination of chronic and acute MI, and volume and pressure overload, appeared consistent and reproducible in each animal. It showed the typical signs occurring during ADHF in patients: impaired LV myocardial function; congestion with increased central venous, pulmonary artery, and pulmonary capillary wedge pressures; pulmonary edema and impaired respiratory gas exchange with systemic acidosis, low arterial PO_2_; and high arterial PCO_2_. Moreover, representative increases in concentrations of circulating cardiac biomarkers such as hs-cTnT, natriuretic peptide, and bio-adrenomedullin occurred, concurrently with the clinical scenario. Finally, elevation of renal and liver biomarkers in the days following onset of ADHF indicated organ injury. Infusion of serelaxin showed results similar to those observed in human patients, namely beneficial effects on congestion, but no effects on mortality [17–20].

### Overview of Currently Available AHF Models in the Large Animal

AHF is a highly prevalent and morbid syndrome, with different etiologic causes, but common pathophysiologic features, including changes in LV structure and function, and neurohormonal activation. Different large animal models of AHF are available, all valuables but with limitations. Thus, an unrestricted literature search was performed on *PubMed* using general keywords, i.e., acute heart failure, heart failure, congestive heart failure, congestion, preclinical models, pig/swine, large animals, and excluding human studies. Authors (FF, LS, RL, GR) selected articles to be discussed, giving priority to those with higher clinical relevance. The ischemia-induced HF is the most commonly used approach [24, 25]. However, acute coronary occlusion often fails to induce stable HF because of the presence of compensatory changes, such as neurohormonal activation, development of a collateral circulation, and LV dilation [10]. Microembolization is another method that has been used to induce ischemic HF, but usually requires multiple injections of microbeads to induce modest cardiac dysfunction [10]. Pressure overload models are less commonly employed due to the complexity of the surgical approach requiring thoracotomy and thus introducing many confounding factors [26]. Volume overload models, induced by valvular regurgitation, produce high LV end-diastolic pressure and dilated cardiac chambers, leading mainly to development of chronic HF and high mortality [27]. Artery to venous fistula models are other methods to induce volume overload HF, but are characterized by a not predictable timing for development of the disease [10, 28]. Tachycardia-induced HF is another non-ischemic approach, which is however reversible with cessation of pacing [10, 29, 30]. Finally, repetitive injections of cardiotoxic drugs are known to produce HF, but require numerous invasive procedures and are affected by high mortality [14]. More model details are in the “supplemental discussion.”

### The Clinical Relevance of the Newly Described ADHF Porcine Model

The new ADHF described in this study may represent, instead, a valid model since it encompasses all the several pathophysiological events occurring during AHF in humans. Thus, an initial MI by occlusion of the LAD caused a chronic HF, represented by the already impaired LVEF at baseline prior to induce the AHF, i.e., < 45%. An AMI additionally induced concurred to a further depress myocardial systolic function with a subsequent decrease in EF, down below 30%. Indeed, a history of ischemic heart disease is present in up to 45–60% of patients with AHF, and nearly 40% of patients with AHF have experienced a prior MI [2, 3, 15, 16, 31–33]. Infusion of a large amount of crystalloids accounted for volume overload, concurring to the development of congestion with significant increases in TFC; presence of extravascular fluid in the lungs and impaired respiratory gas exchange; and raises in mean CVP, PAP, and PCWP to approximately 15, 30, and 40 mmHg, respectively. Volume overload also accounted for significantly increased LVEDV and LVESV, increased LV filling pressures, and worsening of LV systolic function as well as diastolic one, as represented by increased ratio between early mitral inflow velocity and mitral annular early diastolic velocity (*E*/*e′*). Finally, the pressure overload, induced by phenylephrine infusion, contributed to the final decompensation, i.e., through excessive vasoconstriction and consequent increase in afterload [31, 32]. The model was reproducible in each animal and created a condition of ADHF which did not revert when the inductive interventions were stopped but persisted for the whole period of observation. In fact, 48 h later, the signs of congestion ameliorated but LVEF remained impaired, associated with increased LV volumes and low arterial pressure. This model overall reproduced the “wet and warm” (congestion without hypoperfusion) AHF phenotype, the most commonly encountered, i.e., in > 90% of cases, in which pathophysiologically, blood remains upstream of the ventricles, resulting in increased filling pressures and consequently pulmonary congestion and pulmonary edema (left side), whereas congestion of organs in the abdominal cavity, ascites, peripheral edema, or jugular venous dilatation ensues when blood remains upstream of the right ventricle (right side) [3].

The clinical diagnosis of ADHF is supported by circulating biomarkers levels. High LV filling pressure leads to increased ventricular wall stress. Thus, B-type natriuretic peptide (BNP) is secreted from cardiac myocytes in response to atrial or ventricular wall stretch, and its increased levels correlated well with LV function impairment [34]. Thirty to 70% of ADHF patients have also detectable plasma levels of troponins, which are associated with risk of mortality and rehospitalization [35, 36]. Troponin release may be secondary to myocardial injury due to elevated wall stress, direct toxicity of circulating catecholamines, or inflammation [3]. In addition to troponin and natriuretic peptides, elevation of other biomarkers has emerged as predictor of in-hospital mortality, i.e., blood urea nitrogen, serum creatinine, and transaminases are indicative of progression towards the cardio–renal–hepatic syndrome. This syndrome refers to the pathophysiological interplay between the heart and kidney and liver and results in kidney and liver injury and dysfunction. Worsening of renal and liver functions during AHF hospitalization is reported in more than 30% of patients and is associated with high mortality [37–43]. Venous congestion is an essential pathophysiological mechanism of these impaired organ functions, while hypoperfusion accounts for further deterioration. Our model of ADHF resembled the above clinical characteristics. Rapid and significant increases in hs-cTnT, NT-proANP, and bio-adrenomedullin, a novel marker of congestion and AHF [44–46], were observed after onset of ADHF, and such high values persisted over the 2 days of observation. Similarly, at 48 h after ADHF, significant increases in serum creatine and transaminases were observed, as marker of a developed cardio–renal–hepatic syndrome likely contributed to the high mortality observed, i.e., approximately 50%.

### Serelaxin: Testing the New ADHF Porcine Model

Treatment of acute HF remains a major challenge. Diuretics are guideline-recommended first-line therapy in “wet and warm” patients in whom congestion is predominantly attributable to fluid accumulation and volume overload [3, 42, 47]. Vasodilators may be used as first-line therapy to unload the heart and increase venous capacitance in patients with AHF and hypertensive, vascular-type fluid redistribution. Vasodilators improve ventricular function by reducing afterload and decrease symptoms by reducing cardiac filling pressure. In this view, serelaxin has been investigated as potential therapeutic intervention in patients with AHF, showing to improve dyspnea in comparison to standard of care [17–20, 48]. Relaxin is a 6-kDa protein hormone produced by the corpus luteum and secreted into the blood during pregnancy in rodents and humans. Relaxin has been reported to inhibit the stimulation of endothelin-1; modulate the effects of angiotensin; and play anti-inflammatory, extracellular matrix remodeling, angiogenic, and anti-ischemic effects [49, 50].

The Pre-RELAX-AHF trial initially demonstrated a clear vasodilatory effect of serelaxin with trends towards greater resolution of congestion and improved morbidity and mortality in patients admitted for AHF [20]. Subsequently, the RELAX-AHF trial confirmed that a 48-h iv of serelaxin (30 μg/kg per day) in addition to standard of care was associated with significant dyspnea relief but had no effect on readmission to hospital in AHF patients [19]. A 37% reduction in cardiovascular and all-cause mortality was also noted. Unfortunately, a third more recent trial, enrolling 6545 patients who were hospitalized for AHF with the same characteristics of the preceding studies, showed that infusion of serelaxin did not result in a lower incidence of death from cardiovascular causes or worsening HF than placebo [18]. A recent meta-analysis evaluated the effectiveness and safety of 48 h of iv 30 μg/kg/day serelaxin infusion in AHF in six randomized, controlled clinical trials, enrolling a total of 6105 patients randomized to serelaxin and 5254 to control [48]. Administration of serelaxin to patients admitted for AHF was associated with a highly significant reduction in the risk of 5-day worsening HF and in changes in renal function markers. Serelaxin administration was safe and associated with a significant reduction in all-cause mortality.

Serelaxin, through its effects in ameliorating dyspnea and congestion in AHF, represented a valid tool in order to test the accuracy of the proposed new animal model. Indeed, results observed in our model were similar to those obtained in the clinical trials. A beneficial effect represented by a more rapid decrease in lung congestion was observed, i.e., significantly lower TFC, together with a reduction in LV filling pressure and function, with no effects on mortality. In addition, in our model, a more rapid decrease in PAP in serelaxin-treated animals was observed, as in 71 AHF patients receiving serelaxin in whom hemodynamics were invasively investigated [51]. The hemodynamic effects of serelaxin observed in the present study provide plausible mechanistic support for improvement in signs and symptoms of congestion observed with this agent in AHF patients and in our model. Finally, the effects of serelaxin on short-term changes in hs-cTnT, natriuretic peptide, and serum creatinine and transaminases showed significant improvements in AHF patients, consistent with the prevention of organ damage and faster decongestion [19]. Lower serum creatinine, larger mean decrease in ALT, and significantly lower NT-proBNP were reported. Similarly, in our pig model, NT-proANP and adrenomedullin increased significantly after ADHF compared to baseline, but serelaxin was associated with a faster reduction, already after the first hour of infusion. A trend towards a more rapid reduction and/or lower increases in serum creatinine and ALT after serelaxin was also confirmed in our model. Thus, these results suggest that serelaxin reduced cardiac, renal, and liver damage and persistent congestion during the first few days of administration, as observed clinically in AHF patients, further confirming the translational validity of this new model.

### Limitations

Some limitations deserve to be mentioned. The studies were conducted in healthy anesthetized animals and therefore in the absence of underlying diseases or injuries that are causative of AHF and with potential anesthesia-related effects, i.e., cardio-depressant action of propofol and mechanical ventilation-related hemodynamic impact. Second, no data on baseline prior to first AMI were reported. However, hemodynamics, echocardiographic, and biomarker data from healthy pigs from the same breeder, with same age and weight and with the same anesthesia and mechanical ventilation setting, have been previously published by our group [21, 22, 52]. Indeed, a clear reduction in myocardial function was already present 2 weeks after the first AMI in our animals as compared to historical controls [21, 22, 52]. Similarly, the absence of 48-h data from animals dying before the conclusion of the interval of treatment caused a loss of important pathophysiological information. Third, no standard therapy for ADHF was investigated, i.e., diuretics, but only serelaxin was employed as treatment to validate the model. Fourth, the volume overload and pressure overload were acutely induced, without allowing for establishment of long-term compensatory mechanisms and/or neurohormonal responses. Nevertheless, the model was highly reproducible and accounted for all the typical clinical signs occurring during ADHF: impaired LV myocardial function, venous congestion, pulmonary edema, impaired respiratory gas exchange. Representative increases in circulating levels of hs-cTnT, natriuretic peptide, and bio-adrenomedullin were also present, together with increased biomarkers of renal and liver injuries, indicative of cardio–renal–hepatic evolution. Finally, this was an exploratory study, before using this model for formal testing of future potential treatments. For this reason, a formal a priori sample size calculation was not performed. Thus, the number of animals used in the study was not adequate to attain a reasonable statistical power to compare serelaxin vs. saline infusion, but suitable to provide preliminary results and indications about the validity and reproducibility of the model for planning future experiments. Indeed, administration of serelaxin confirmed the validity of this model, showing results similar to those reported in clinical studies, i.e., a trend towards a beneficial effect on congestion with organ protection as represented by circulating markers, but no effects on mortality [17–20].

## Conclusions

Several animal models of AHF have been developed and employed over the years, all valuables but with limitations in reproducing disease mechanisms and pathophysiology. A new model of ADHF, resulting from a combination of chronic and acute MI, and volume and pressure overload, appeared able to replicate all the clinical features of the disease. The proposed model may represent a reproducible and valid approach to test new treatments for AHF prior to being clinically translated.

## Electronic Supplementary Material

ESM 1 (DOCX 526 kb)

ESM 2 (DOCX 14 kb)

ESM 3 (AVI 33753 kb)

ESM 4 (AVI 32346 kb)

ESM 5 (AVI 42190 kb)

ESM 6 (AVI 40784 kb)

## Data Availability

Data are available upon request.
