# Supplementary material for: Searching for Preclinical Models of Acute Decompensated Heart Failure: a Concise Narrative Overview and a Novel Swine Model
Source: Cardiovasc Drugs Ther. 2020 Oct 24;36(4):727–38. doi: 10.1007/s10557-020-07096-5 (PMC9270312; doi:10.1007/s10557-020-07096-5)

**Supplemental Figure 1.**

Osmotic infusion pump (Azlet®) implantation for the 48-hr continuous iv infusion of serelaxin or saline placebo. The pump was surgically positioned and secured with sutures between the right sternocleidomastoid muscle bands. The polyethylene catheter was inserted into the right external jugular vein and advanced for 8 cm.


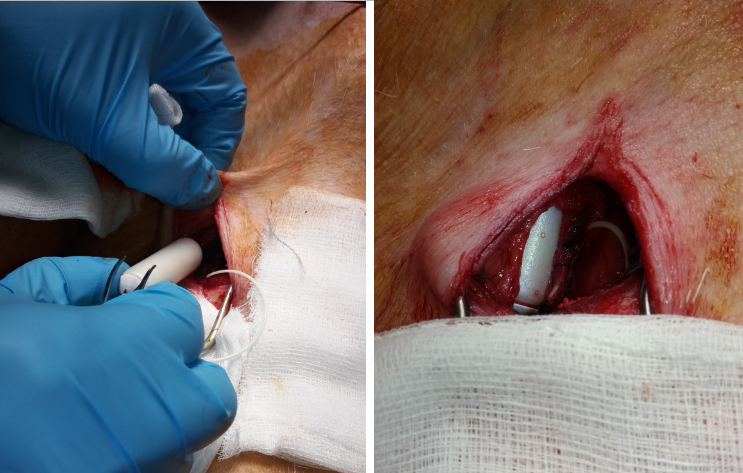

Supplement: Supplementary file 1 — (DOCX 526 kb) [file 10557_2020_7096_MOESM1_ESM.docx]
