# Supplementary material for: Searching for Preclinical Models of Acute Decompensated Heart Failure: a Concise Narrative Overview and a Novel Swine Model
Source: Cardiovasc Drugs Ther. 2020 Oct 24;36(4):727–38. doi: 10.1007/s10557-020-07096-5 (PMC9270312; doi:10.1007/s10557-020-07096-5)
Supplement: Supplementary file 2 — (DOCX 14 kb) [file 10557_2020_7096_MOESM2_ESM.docx]

**Supplemental Discussion**

*Currently available AHF models in the large animal: a concise narrative overview*

AHF is a highly prevalent and morbid syndrome, with different etiologic causes, but common pathophysiologic features, including changes in LV structure and function, and neurohormonal activation. Different large animal models of AHF are available, all valuables but with limitations. The ischemia-induced HF is the most commonly used approach for its reproducible induction of systolic dysfunction through occlusion or ligation of the LAD or LCx [24, 25]. Acute coronary occlusion depresses LV function and prompts neurohormonal activation, thus satisfying several criteria characteristic of the AHF condition. However, acute coronary occlusion often fails to induce stable HF because of the presence of compensatory changes, such as neurohormonal activation, development of a collateral circulation, and LV dilation [10]. To make this model less relevant compared to others is also the high degree of variability in myocardial ischemia resulting from the coronary artery occlusion, due to differences in the pattern of distribution of the coronary artery and presence of collateral backup. Microembolization is another method that has been used to induce ischemic HF [10]. This method, however, usually requires multiple injections of microbeads to induce modest cardiac dysfunction. Large animal models of pressure-overload are less commonly employed compared to the ischemic ones. They consist of surgically applied banding in the ascending aortic, that produces a hemodynamic profile similar to that observed in patients with aortic stenosis [26]. However, the banding technique is complex and requires thoracotomy, adding confounding factors in outcome. In volume-overload models, inducted by valvular regurgitation, left-to-right cardiac or vascular shunt, and anemia, overloading the heart with excessive volume promotes cardiac dysfunction. Increased volume load, in fact, results in high LV end-diastolic pressure and dilated cardiac chambers. Both aortic and mitral valves can be targeted to induce volume-overload, but the mitral valve regurgitation by a catheter-based method of chordae disruption is the most used approach. This model, however, leads mainly to development of chronic HF, and is accompanied by high mortality, related to the difficulty in controlling the degree of regurgitation [27]. Placement of inferior vena cava filters or creation of large LV posterior wall infarction represent other reported approaches, although their validity is disputable [10, 24]. Artery to venous fistula models are other methods to induce volume-overload HF [28]. However, the time course for the development of HF is not predictable, while a laparotomy is necessary to expose the abdominal inferior vena cava and aorta to create a shunt or fistula [10]. Tachycardia-induced HF is another well-established and reproducible non-ischemic model [10, 29, 30]. Rapid pacing of the atrium or the ventricle for 4-8 weeks leads to elevation of LV end-diastolic pressure accompanied by systolic dysfunction, that is, however, reversible with cessation of pacing [30]. Moreover, this model failed to demonstrate the true underlying mechanisms of AHF [11]. Cardiotoxic drug injections into the coronary artery also result in systolic dysfunction with fibrosis and myofiber atrophy [14]. However, this approach is associated with high mortality and requires repetitive invasive administration of drugs.

[*References in the main manuscript*]
